# Trends in vascular pharmacology research in the Department of Pharmacology and Clinical Pharmacology, Faculty of Medicine, Comenius University, Bratislava

**DOI:** 10.2478/v10102-011-0008-8

**Published:** 2011-03

**Authors:** Viera Kristová, Milan Kriška, Róbert Vojtko, Miriam Petrová, Silvia Líšková, Radoslav Villáris, Zoltán Varga, Martin Wawruch

**Affiliations:** Department of Pharmacology and Clinical Pharmacology, Faculty of Medicine, Comenius University, Bratislava, Slovak Republic

**Keywords:** vascular responses, models of endothelial damage, computer-based studies, cardiovascular drugs, NSAIDs, adverse drug effects

## Abstract

Research in the Department of Pharmacology started to focus intensively on fetal circulation in the 60s. Results of experiments contributed to clarification of the conversion of fetal circulation type to the adult type: the mechanism of the ductus arteriosus closure, examination of fetal and neonatal pulmonary vessel responses. In the early 80s, increased attention was dedicated to fetal vascular endothelium, later on to vascular reactivity in relation to the endothelium in adult animals. We developed original models of vascular endothelial damage using the perfusion method (repeated vasoconstrictive stimuli, deendothelization by air bubbles). We developed a new technique for *in vitro* endothelial loss quantification on Millipore filters. Under *in vitro* conditions, the protective effects of sulodexide and pentoxifylline on vascular endothelium were evaluated. In recent years were studied protective effects of selected substances *in vivo* in models of endothelial damage (*e.g.* stress, toxic tissue damage, diabetes mellitus, hypertension). The role of potassium channels in the hypertension model was studied in cooperation with the Czech Academy of Sciences. Assessment of vascular reactivity in the diabetic model was significantly improved by computer. In addition to experimental work, the department is solving problems of clinical pharmacology – especially drug risk evaluation (non-steroidal anti-inflammatory drugs). Recently, we have dealt with pharmacoepidemiological studies in geriatric patients and with cardiovascular risk of NSAIDs in relation to pharmacotherapy. The results of these studies may be an impulse for targeted problem solving in our experiments.

## Introduction

Research in cardiovascular pharmacology has a long tradition in our department and in the past decade it has undergone significant changes. In the 60s, we focused on fetal blood vessels, and particularly on the role of fetal shunts, such as the ductus arteriosus, in the conversion from a fetal to an adult type of circulation. Research achievements of Kovalčík (1963) contributed significantly to the clarification of the mechanism of ductus arteriosus closure and especially of the crucial role of oxygen in this closure. The role of prostaglandins, kinins and other vasoactive substances, as well as the myogenic activity of ductus arteriosus, was studied in other research work of the department (Kriška & Kovalčík, 1973; Smieško *et al*., 1978). With time, the topic of fetal vessels logically continued in the study of pulmonary and umbilical vessels reactivity (Kristová *et al*., 1986), which in the perinatal period contributes crucially to the transformation of fetal circulation to the adult type. In addition to the study of effects of vasoactive substances (biogenic amines, kinins, prostaglandins) and medicaments (ambroxol, glucocorticoids) on pulmonary vessels, research was extended to evaluate effects of endothelin on the pulmonary circulation of fetal sheep (Cassin *et al*., 1991). Parallel with the research of vessel reactivity in ontogenesis, our department also examined the effects of hormones on vascular responses. In the early 80s, increased attention was given to fetal vascular endothelium, starting from the morphological aspect and later on to the vessel response (Babál *et al*., 1987). Subsequently, we focused our study on vascular reactivity in relation to the endothelium in adult animals (Kriška *et al*., 1989). We evaluated the impact of factors increasing the risk of endothelium damage – as the duration of vessel storage and application of vasoconstrictive substances (Kristová *et al*., 1993). Results of these experiments led to their targeted use in further experiments. We designed in vitro models of vascular endothelial damage using the perfusion method – method of repeated vasoconstrictive stimuli induced by norepinephrine, vascular deendothelization by air bubbles, and endothelial damage by perfusion with activated polymorphonuclear leukocytes. Under conditions of the perfusion method, we developed an original technique to quantify the endothelial loss on Millipore filters after the use of deendothelization stimuli (Babál *et al*., 1992).

After verification of experimental models of endothelial damage in vitro, we started to evaluate effects of different drugs and substances on the endothelium. First we investigated effects of nonsteroidal anti-inflammatory drugs, which by inhibiting the synthesis of vasodilator prostaglandins potentiated the vasoconstrictive responses of femoral and renal arteries (Kristová *et al*., 2000, 2002).

Under conditions *in vitro*, the potential protective effect of sulodexide and pentoxifylline on vascular endothelium was evaluated (Kristová *et al*., 1995, 2000; Babál *et al*., 1996). In the last years, we studied endothelium-protective effects of selected substances in experimental models of endothelial damage *in vivo*; particularly in the stress model, the model of toxic tissue damage by carbon tetrachloride and in the diabetic model (Kristová *et al*., 2006, 2008; Babál *et al*., 2006). Mechanism of vascular reactivity in relation to potassium channels in hypertensive animals were revealed in experiments carried out in cooperation with the Institute of Physiology, Academy of Sciences, Czech Republic (Líšková *et al*., 2007). The results obtained by traditional methods of vascular response evaluation often fail to show an authentic view on contractile responses (phasic and tonic component). Digital recording of contractile responses using modern software enables mathematical analysis of perfused vessel reactivity. We obtained original results, which quantify a wide set of parameters not investigated so far (Dedík *et al*., 2003; Vojtko *et al*., 2010).

In cooperation with clinical institutes, our department has been dealing for a long time with some problems of clinical pharmacology – mostly based on experimental results. As the most serious problem we consider drug risk evaluation, concerning particularly analgesics. Adverse effects of nonsteroidal anti-inflammatory drugs in high-risk patients were evaluated in cooperation with National Institute of Rheumatic Diseases. In collaboration with clinical departments, problems of pharmacotherapy of peripheral circulation disorders were studied (Kriška *et al*., 2002; Rajec *et al*., 2007).

Recently, the staff of our department has focused on the pharmacoepidemiological studies in geriatric patients (Wawruch *et al*., 2008) and on the cardiovascular risk of NSAIDs in relation to pharmacotherapy (Varga *et al*., 2010). The results of these studies may be an impulse for targeted problem solving, especially in clarifying serious cardiovascular disorders resulting from drug interactions.

## Selected results from the recent period

## A. Experimental part

### Evaluation of endothelium-protective effects of sulodexide on experimental model of diabetes mellitus

Diabetes mellitus (DM) is generally associated with many cardiovascular complications accompanied with development of endothelial dysfunction or damage. Therefore, the usage of some drugs, as statins, ACE inhibitors, pentoxifylline, may have a protective effect on the endothelium. Based on our previous experience with sulodexide (glycosaminoglycan composed of heparin-like and dermatan fractions), we decided to evaluate its endothelium-protective effects in the model of streptozotocin-induced DM. We focused mainly on measurement of the number of circulating endothelial cells in peripheral blood as a morphological marker of endothelial injury and on evaluation of acetylcholine-induced relaxations of the rat mesenteric artery as a marker of endothelial function.

#### Methods

The following groups of animals were used in experiments: control (C, saline solution), sulodexide (SLX, 100 UI/kg/day), DM, induced by administration of streptozotocin (STZ in the dose 30 mg/kg/day i.p. over 3 consecutive days), DM+SLX. Treatment with sulodexide lasted 10 weeks. Isolated vessels were cut into rings for measurement of isometric contractions. The number of endothelial cells in blood was measured in Bürker′s chamber according to a previously described method (Kristová *et al*., 2006).

#### Results

**Figure 1:**
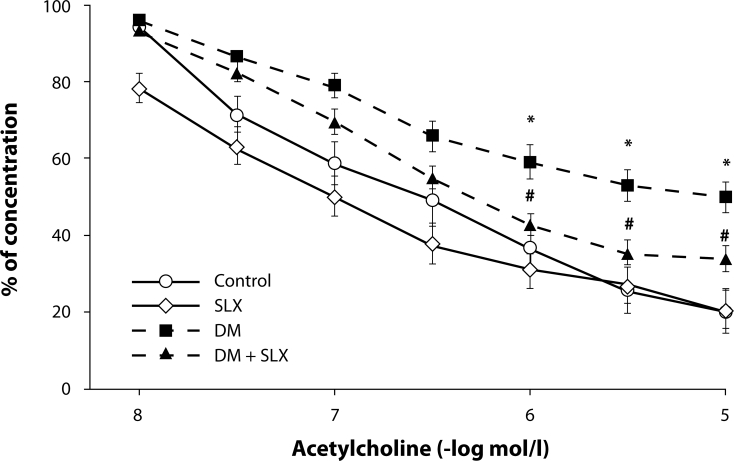
Effect of sulodexide treatment (SLX) on acetylcholineinduced relaxation of mesenteric artery in rats with 10-week diabetes (DM) compared to control and diabetic rats treated with sulodexide (DM+SLX). ^*^p<0.05: Control vs. DM, ^*#*^*p<0.05:* DM vs. DM+SLX.

Ten weeks of diabetes significantly impaired acetylcholine-induced relaxations of isolated mesenteric arteries compared to controls (Figure 1). SLX treatment improved endothelium-dependent relaxation in mesenteric arteries although the control level was not reached. Increased endothelemia was found in diabetic animals compared to the controls, which is in agreement with results of our previous study (Zúrová-Nedelčevová *et al*., 2006). SLX significantly decreased the number of circulating endothelial cells (Table 1). The obtained results demonstrate that sulodexide has endothelium protective effects in streptozotocin-induced diabetes. The favorable effects of SLX in peripheral occlusive arterial disease were confirmed in clinical studies (Gaddi *et al*., 1996).

**Table 1:** Effect of sulodexide (SLX) on number of circulating endothelial cells in plasma [EC/10µl] in control (C), SLX treated (SLX), diabetic (DM) and diabetic rats treated with SLX (DM+SLX) in 10-week diabetes mellitus.

C	SLX	DM	DM+SLX
**1.88**	**2.38**	**3.88**	**2.25**
(0.75; 2.25)	(1.75; 3.00)	(3.25; 5.75)****^++^	(1.75; 3.50)^#^

Data are expressed as medians and their 95% confidence intervals, ***p<0.0001: Control vs. DM, ^++^ *p*<0.01: SLX vs. DM, ^#^ *p*<0.02: DM vs. DM+SLX.

### Effects of L-NNA and indomethacin on vascular tone of femoral arteries in Wistar-Kyoto and spontaneously hypertensive rats

Prevention and treatment of hypertension have been intensively studied, but the mechanisms of hypertension remain still elusive. Hypertension is the leading risk factor of cardiovascular diseases. Cardiac output and peripheral resistance regulate blood pressure. Our group focuses on the regulation of vascular tone, which determines peripheral resistance. There are several relaxing and constricting factors, mostly produced by the endothelium, which control vessel diameter, blood flow and vascular tone. The main vasodilating factors produced by the endothelium are nitric oxide (NO), endothelium derived hyperpolarizing factor (EDHF) and prostaglandins (PGI_2_, PGE_2_, PGD_2_). The basic vasoconstrictors include endothelins, thromboxane A_2_ (TXA_2_) and prostaglandins (PGG_2_, PGH_2_). Endothelium-derived constricting factor (EDCF) is probably a product of cyclooxygenase activity, but this factor has not been identified until today.

#### Methods

Our experiments are oriented on the effect of endothelium-derived relaxing (NO) and constricting (EDCF) factors in norepinephrine (NE)-induced contraction. Isolated femoral arteries of 6-month-old normotensive Wistar-Kyoto rats (WKY) and 6-month-old spontaneously hypertensive rats (SHR) were placed in on isometric Mulvany-Halpern myograph. Contraction of femoral arteries with intact endothelium were induced by cumulative doses of NE (10^−9^–10^−5^ mol/l). The presence of endothelium was tested by acetylcholine application. NE-induced contraction response curves were constructed under control conditions as well as in the presence of the NO synthase inhibitor N(ω)-nitro-L-Arginine (L-NNA; 10^−4^ mol/l) and the cyclooxygenase inhibitor indomethacin (IME; 10^–^5 mol/l).

#### Results

**Figure 2:**
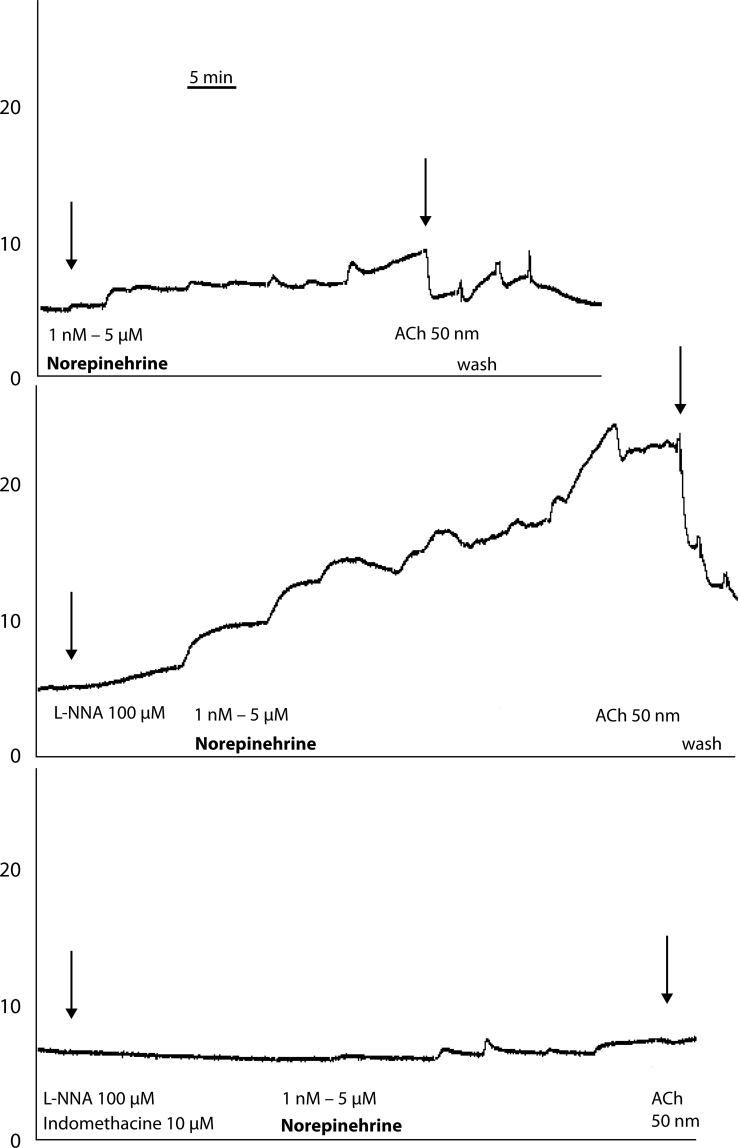
Reactivity of isolated femoral artery with intact endothelium (original records from myograph), Wistar-Kyoto rats.Horizontal axis: time in min. Vertical axis: wall tension in mN/mm.Ach – acetylcholine, arrows indicate application of initial norepinephrine or acetylcholine dose. At the top: norepinephrine-induced contraction. In the middle: norepinephrine-induced contraction in the presence of L-NNA (N(ω)-nitro-L-Arginine). At the bottom: norepinephrine-induced contraction in the presence of L-NNA and IME (indomethacin).

Our results showed that the NE-induced contraction was augmented in vessels isolated from hypertensive animals. The application of L-NNA increased NE-induced contraction in WKY (Figure 2), but was almost without effect in vessels from SHR. Inhibition of cyclooxygenase markedly impaired NE-induced contractions of femoral arteries of WKY (Figure 2) and SHR.

This impairment was more pronounced in arteries of SHR. We suggest that one possible mechanism of enhanced contractility of vessels during hypertension may be explained by the action of endothelium-derived constricting factor.

### Contribution of computer-based modeling to assessment of vascular reactivity of diabetic rats

Evaluation of contractile responses of perfused vascular segments in vitro is traditionally based mainly on determination of the maximum response – amplitude (*p*_*max*_) and in some cases on the time needed from the start of the record to reach this peak (*t*_*max*_). Such descriptive methods of evaluation are related to data extraction from graphic records of contractions obtained by analog recorders and loaded with subjective inaccuracy of evaluator and a lower reproducibility of the acquired data.

Substantial enrichment of the results of our experimental work was provided by the involvement of new software, using the measurement card recording the contraction response of isolated segments digitally. This enabled quantification and evaluation of a wide set of contractile response parameters, whose determination was not possible by routine descriptive methods. Digital recording of the vessel segment responses to series of succesively increasing doses of norepinephrine was followed by computational modeling determining the resulting parameter values of the segment reactivity. In comparison with analog data extraction, no information gets lost – two key parameters measurable by descriptive procedures (*p*_*max*_, *t*_*max*_) quantify computer methods more rapidly and more precisely.

#### Methods

Computer-based modeling of digital records of contractions is based on general system theory and uses the analysis of the properties of the system tested through modeling output responses (contractions) to defined inputs (single doses of norepinephrine). The initial step of this analysis is a data-number reduction by standard algorithm, which from originally recorded approximately 250 points retains 30–40 representative points for the course of every contractile response. For the next analysis each artery segment is considered as dynamic system *H* and computer-based modeling determines a representative set of model parameters, as defined by the system transfer function *H*(*s*):

$$H(s)=G\frac{a_{0}+a_{1}s+a_{2}s^{2}+\ldots +a_{n}s^{n}}{1+b_{1}s+b_{2}s^{2}+\ldots +b_{m}s^{m}}$$,

where *G*, *a*_*0*_, …. *a*_*n*_, *b*_*1*_, …. *b*_*m*_ are the parameters of the model, *n* and *m* are the highest values of numerator, respectively denominator of the polynomial *H*(*s*). The values of the transfer function as an argument of complex variable *s*, represent the ratio of the output and input of system *H* after the Laplace transformation. The modeling procedures used were described by Dedík *et al*. (2003). The final result of modeling is to obtain the representative model curves of contractile responses and quantification of defined model parameters. These include the most important and characteristic ones for the contractile response – vascular sensitivity *S* (determined by the parameter *G*, in our model *S*=*G*), mean time of vasoconstrictor response *MTVR* and the rate constant of relaxation *k*_*rel*_.

Sensitivity of the renal artery segment determines the ratio of the area under curve of the measured profile of contractile response and norepinephrine dose. Mean time of vasoconstrictor response represents the time point of centroid of this profile. Rate constant of relaxation stands for the lowest from the absolute values among the members in the denominator of the polynomial *H*(*s*) equation. The sensitivity *S* in relation to the effect of norepinephrine represents its intensity, *MTVR* its duration and *k*_*rel*_ its regression (Dedik *et al*., 2003).

#### Results

**Figure 3:**
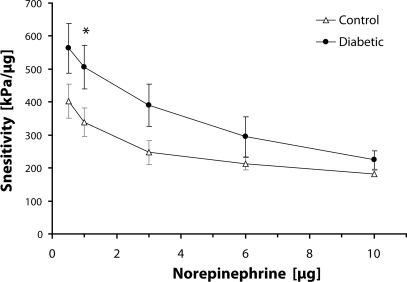
Sensitivity of renal artery segments. Comparison between control group and diabetic. Values are given as mean± SEM, *p<0.05.

Our results in the model of streptozotocin-induced diabetes in rats were obtained by application of the computer-based modeling procedures for detailed data extraction. On the one hand, values of descriptive parameters *p*_*max*_ and *t*_*max*_ did not show statistically significant differences in the reactivity of renal artery segments between the control and diabetic group. On the other hand, the course of the curves showing values of the key parameter *S* for each dose of norepinephrine (range 0.5–10 µg) showed distinct differences with statistical significance at the dose of 1 µg (Figure 3).

**Figure 4:**
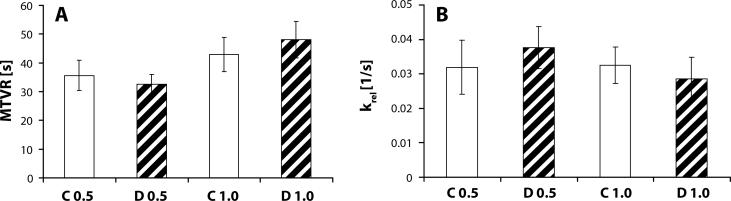
Mean time of vasoconstrictor response (**A**) and the rate constant of relaxation (**B**) of renal artery segments at norepinephrine doses 0.5 and 1.0 µg. C 0.5 – control group, dose 0.5 µg; C 1.0 – control group, dose 1.0 µg; D 0.5 – diabetic group, dose 0.5 µg; D 1.0 – diabetic group, dose 1.0 µg. Values are given as mean ± SEM.

Evaluation of parameters *MTVR* (Figure 4A) and *k*_*rel*_ (Figure 4B) for selected doses with the highest sensitivity at 0.5 and 1 µg and the model type 1–4 (*n*=1, *m*=4) showed difference between the control and diabetic group, but without statistical significance. The model parameter *S* and *k*_*rel*_ values suggest a slower pressure decrease in the vascular segment after reaching the peak value in the diabetic group compared to control. Based on these results, we expect disturbance of the wall elasticity of the renal artery segments tested which may increase vascular rigidity (Vojtko *et al*., 2010). Such alterations associated with diabetic angiopathy have been documented by several clinical and experimental studies (Lagaud *et al*., 2001; Sivitz *et al*., 2007). Computational modeling as a methodological approach shows a new previously unavailable way to precise evaluation of elasticity and other essential functional properties of the vessel wall using the perfusion method, which belongs to *in vitro* methods closest to physiological conditions.

The described mathematical modeling of digitally recorded contractile responses meets the expectations of accuracy and high reproducibility of data extraction, representing an improvement of the traditional assessment of analog records. It may offer a new view by providing a more detailed and exact description of responses to different substances on vessel segments, which seems to be a significant progress compared with routine descriptive methods.

## B. Clinical part

### Cardiovascular drugs potentially inappropriate for elderly patients

Persons aged ≥65 years are more susceptible to adverse drug reactions compared to younger people. Changes in drug pharmacokinetics and pharmacodynamics, frequent non-compliance, polymorbidity and polypharmacy contribute to a greater frequency of drug-related problems (Turnheim, 2003). Higher occurrence of adverse effects in elderly patients is associated particularly with the administration of certain drug. Therefore several lists of potentially inappropriate medications for elderly patients were published at the end of the 20^th^ and the beginning of the 21^st^ century (McLeod *et al*., 1997; Fick *et al*., 2003). These lists include drugs with increased risk of adverse effects if they are administered to patients aged 65 or more. Other reasons for including a medicine into such lists are high probability of drug-drug interaction and lack of information on the efficacy of the drug in elderly patients in accordance with the requirements of evidence-based medicine. The Beers criteria published in 2003 represent an internationally recognized list of potentially inappropriate medications and they reflect current knowledge in the field of gerontopharmacology.

#### Methods

The sample of our study (n=345) was selected from 524 patients aged ≥65 years hospitalized in a municipal hospital during the time period of August 1, 2004 to March 31, 2005. Patients who died during hospitalization and those with incomplete documentation were excluded from the study. All medications at the time of hospital admission were recorded for each patient. Cardiovascular medications potentially inappropriate for older patients were identified using modified Beers criteria published in 2003 (Fick *et al*., 2003).

#### Results

The mean age of the whole group (n=345) was 76.9±7.4 years. The study population consisted of 206 (59.7%) women and 139 (40.3%) men. At the time of hospital admission, 65 (18.8%) out of 345 patients were treated with at least one potentially inappropriate medication (Table 2). The most frequently prescribed potentially inappropriate medicine was digoxin at a dose >0.125 mg/day (except when treating atrial arrhythmias), followed by ticlopidine and amiodarone. The high use of digoxin at doses>0.125 mg/day may be explained by the frequent occurrence of chronic heart failure in the evaluated group as well as by remaining prescription habits. Ticlopidine was the most frequently prescribed potentially inappropriate medication also in the study of Onder *et al*. (2005). The authors explain this finding by unavailability of the safer alternative clopidogrel in Italy at the time of their analysis (1997–1998). Similarly the prescription limitation caused by the high price of clopidogrel contributed to the high prevalence of ticlopidine at the time when our study was carried out. The situation is changing due to current availability of cheaper generic variants of clopidogrel.

**Table 2:** Potentially inappropriate cardiovascular drugs used in treatment of elderly patients.

Drug	Frequency (% of n=345)
digoxin*	44 (12.8)
ticlopidine	28 (8.1)
amiodarone	19 (5.5)
methyldopa	5 (1.4)
doxazosin	3 (0.9)

digoxin in doses >0.125 mg/day (except when treating atrial arrhythmias)

Rational attempts to reduce the use of potentially inappropriate medications can have a positive impact on the healthcare system. Our results underline the importance of paying more attention to the topic of pharmacological treatment in elderly patients during pre- and post-graduate education of healthcare professionals.

### Use of non-steroidal anti-inflammatory drugs and risk of cardiovascular adverse effects in hospitalized patients

The group of non-steroidal anti-inflammatory drugs (NSAIDs) is one of the most prescribed drug groups worldwide. Several NSAIDs are available over the counter; they are used by a large number of patients without prescription. The widespread use of non-steroidal anti-inflammatory drugs is caused by the high prevalence of diseases causing acute or chronic pain in the population. Diseases accompanied by inflammation and fever also occur in a substantial number of patients. Adverse drug reactions of NSAIDs represent a serious medical problem because of the high number of exposed patients. Continuous analysis of analgesic usage is needed to improve the safety of analgesic therapy.

#### Methods

Retrospective data collection from documentation of patients hospitalized in the department of internal medicine of a district hospital; data were analyzed using methods of descriptive statistics.

#### Results

In our recent pilot study, data obtained from medical records of 112 patients receiving NSAIDs during hospitalization in the department of internal medicine were evaluated with focus on analgesic prescription and risk of cardiovascular adverse effects (Varga *et al*., 2010). The mean age was 63.7 years (SD, 15.0), 67% of the patients in the study group were women. The most commonly used non-steroidal anti-inflammatory drug was metamizol (76patients; 67.9% of the study group). Diclofenac (20; 17.9%), indomethacin (16; 14.3%) and ketoprofen (14; 12.5%) were also used by a high number of patients. Preferential inhibitors of COX-2 were administered to 17 patients (nimesulide – 10; 8.9%; meloxicam – 7; 6.3%). Arterial hypertension, chronic heart failure and ischemic heart disease were the most frequent diagnoses indicating increased risk for development of cardiovascular adverse effects of NSAIDs (Table 3).

**Table 3:** Diagnoses indicating increased risk of CVS adverse effects.

Diagnoses	Number of patients	% of the study group
Arterial hypertension	64	57.1%
Chronic heart failure	47	42.0%
Ischemic heart disease	46	41.1%
Diabetes mellitus	33	29.5%

**Figure 5:**
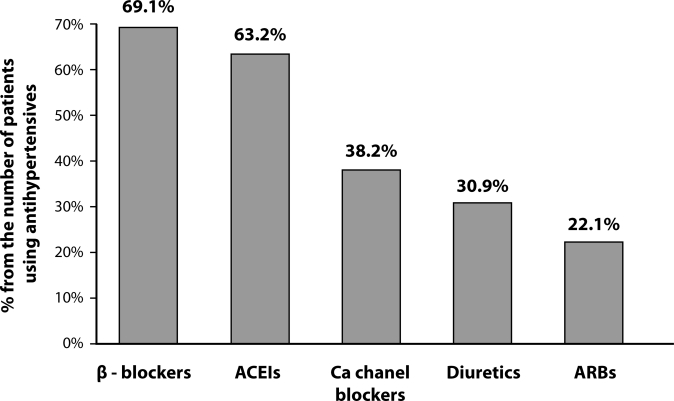
Drugs used in antihypertensive treatment in study group.

Administration of NSAIDs can lead to elevation of arterial blood pressure (Chan *et al*., 2009; White, 2007), the greatest risk is present in patients with controlled arterial hypertension using ACE inhibitors, angiotensin receptor blockers and beta-blockers (White, 2007). In our study group, the most frequently used antihypertensive drugs were beta-blockers and ACE inhibitors (Figure 5).

Results of the discussed pilot study need to be confirmed in a study with a large number of enrolled patients. The possible pathophysiological mechanisms leading to cardiovascular toxicity of NSAIDs will be studied in animal experiments.
